# Exploring the knowledge level and practices of hospital pharmacists in management of oral anticoagulants in Gulf Cooperation Council countries: a scoping review of literature

**DOI:** 10.3389/fpubh.2026.1743611

**Published:** 2026-01-22

**Authors:** Abdulaziz Alanazi, Atta Abbas Naqvi, Nilesh Patel, Francesco Tamagnini

**Affiliations:** 1 School of Pharmacy, University of Reading, Reading, United Kingdom; 2 Institute of Pharmaceutical Science, King's College London, London, United Kingdom

**Keywords:** counselling, direct oral anticoagulants, GCC countries, knowledge, pharmacists, practice, warfarin

## Abstract

**Introduction:**

Oral anticoagulants (OACs) are essential for managing thromboembolic events and cardiovascular conditions. However, they carry a significant bleeding risk. Pharmacists play a critical role in ensuring the safe and effective use of these medications. Within Gulf Cooperation Council (GCC) countries, hospital pharmacists are involved in managing OACs. However, region specific data on their knowledge and practices remain limited.

**Aim:**

This review analysed existing literature regarding hospital pharmacists’ knowledge and practices in OACs dispensing and counselling within the GCC countries.

**Methodology:**

A literature search was conducted in Scopus, Web of Science, PubMed and PsycINFO. Articles that explored the hospital pharmacist’s knowledge and practices regarding OACs were included. There were no restrictions on study design, publication date, or language. Searches were undertaken on February 1, 2025 then re-run on October 28, 2025, following PRISMA-ScR and JBI guidelines.

**Results:**

Findings revealed gaps in pharmacists’ knowledge and practices regarding OACs management. Out of 75 articles identified, seven met inclusion criteria, representing studies from Bahrain, Kuwait, Oman, Qatar, Saudi Arabia, and the United Arab Emirates (UAE). All were cross-sectional and utilized validated questionnaires. A study from Saudi Arabia and another from the UAE reported gaps in warfarin knowledge. Counselling practices were suboptimal in both studies. One study further identified insufficient knowledge of warfarin interactions. Beyond warfarin, one study reported deficiencies in rivaroxaban knowledge and counselling, while another found moderate awareness of direct oral anticoagulants (DOACs) (mean score 41.6% ± 26%). Another study reported inadequate practice in OACs dispensing and monitoring, and another study found greater pharmacists’ confidence in counselling on vitamin K antagonists (VKAs) (67%) than on DOACs (49%).

**Discussion:**

Findings highlight inadequate knowledge and practice among pharmacists regarding OACs. Although all studies included community pharmacists and other healthcare providers, none focused exclusively on hospital pharmacists, who are more directly involved in OAC management in the GCC. All studies relied solely on self-reported data, increasing bias. Most studies originated from Qatar, Saudi Arabia, and the UAE, reducing generalizability. Nevertheless, addressing knowledge gaps could improve hospital pharmacists’ practice on OACs management, improving patient safety, and optimising therapeutic outcomes.

## 1 Introduction

Oral anticoagulants are medications that prevent blood clot formation by inhibiting the coagulation cascade, thereby prolonging clotting time (1). OACs include vitamin K antagonists such as warfarin, as well as DOACs such as dabigatran, rivaroxaban, and apixaban (1). They are commonly prescribed for the treatment of venous thromboembolism (VTE) and the prevention of thromboembolic events in conditions such as atrial fibrillation and myocardial infarction (1). Despite their clinical benefits, OACs have been classified as high-alert medications, and are associated with serious patient harm when used inappropriately (2).

Pharmacists, as medication experts, are central to achieving safe and effective use of OACs (3). Their role in use of OACs varies globally depending on the healthcare setting and the specific medicine used (3). Within hospital settings, pharmacists perform complementary roles as inpatient, outpatient, or clinical pharmacist, where each role characterised by distinct scopes of practice and levels of direct patient care (4). However their responsibilities, generally, extend beyond dispensing to include prescription review to ensure appropriateness and adherence to clinical guidelines, educating patients and caregivers, renal dose adjustments, monitoring for drug interactions, and advising on therapy switches based on clinical status (4). Hospital pharmacists in particular play a central role in optimizing OACs therapy and improving patients clinical outcomes.

Evidence from a systematic review of randomized controlled trials demonstrated that pharmacist-led interventions improved medication adherence and clinical outcomes, highlighting their essential role in ensuring the safe and effective use of anticoagulant therapy (5). Similarly, the systematic review by Manzoor and colleagues demonstrated that pharmacist-managed outpatient anticoagulation services consistently achieved higher quality of anticoagulation control and are associated with fewer bleeding, thromboembolic events, and reduced hospitalizations (6). Moreover, discharge education provided by pharmacists has been shown to enhance patients’ understanding and promote appropriate anticoagulant use (7). Consistent with these findings, a recent systematic review and meta-analysis further confirmed that pharmacist-led interventions were associated not only with greater appropriateness of anticoagulant therapy but also with significant reductions in bleeding complications and hospital readmissions (8). Collectively, these studies underscore the value of pharmacists as integral members of the healthcare team in optimising anticoagulation outcomes.

Given the high burden of cardiovascular diseases and thromboembolic events, the need for OACs has steadily increased. Stroke alone affected 93.8 million individuals and caused approximately 7.25 million deaths in 2021 worldwide (9). Atrial fibrillation (AF), another major indication for OAC therapy, impacted 52.6 million people worldwide and contributed to 339,000 deaths (10). Venous thromboembolism also represents a significant public health challenge, with an estimated 10 million cases annually; within this, deep vein thrombosis (DVT) affects more than 465,000 individuals in the European Union and over 300,000 in the United States each year (11). Reflecting this growing clinical need, oral anticoagulant drug expenditure and prescribing trends have risen sharply. In the UK, the NHS reported issuing more than 24 million direct oral anticoagulant prescriptions between 2021 and 2023 (12). Whereas oral anticoagulant expenditure in the United States increased from $3.4 billion in 2014 to $17.8 billion in 2020 (13).

A similar pattern is evident in the GCC region where diseases requiring OACs is rising. A systematic review looking at adult populations in the Gulf region reported a 5.5% prevalence of cardiovascular diseases (14). Specifically, stroke accounted for around 34,900 deaths and an estimated economic loss of $5.11 billion in the GCC (15). In Saudi Arabia, ischaemic heart disease and stroke are among the leading causes of mortality with approximately 1.6% of adults affected (16, 17). Given this high disease burden, the demand for OACs, particularly DOACs, has risen sharply in the GCC (18). It is projected that by 2025, expenditure on anticoagulants in the GCC will reach $308.65 million, with Saudi Arabia accounting for $181.85 million of this total (19). Together, these figures highlight the growing reliance on OACs emphasizing the need for effective OACs management to improve patient outcomes.

The healthcare system in the GCC operates as a mix of public and private providers. Within the public sector, the Ministry of Health holds primary responsibility for funding, regulating, and managing healthcare facilities (20). However, the public healthcare system remains fragmented, as additional providers, such as military hospitals, offer services exclusively to their employees (21). On the other hand, the private sector delivers healthcare services either on a charge or through medical insurance coverage. Its funding is primarily derived from employer contributions, out-of-pocket payments, and private health insurance (22). In this context, healthcare systems within the GCC countries namely Saudi Arabia, United Arab Emirates, Bahrain, Qatar, Kuwait, and Oman are undergoing substantial transformation aimed at improving care delivery (18). One area of particular importance in this transformation is the management of cardiovascular and thromboembolic diseases, which pose a major public health challenge in the region.

One of the most effective strategies to prevent complications associated with OACs is patient education, which in turn depends on hospital pharmacists possessing up-to-date and comprehensive knowledge of these medications (23). Educating patients about their prescribed medicines including the correct dose, frequency, potential side effects, and drug or food interactions as well as the importance of adherence. Effective education not only informs patients of the therapeutic benefits but also guide them on when to seek medical advice, thereby reducing preventable adverse events (24). Additional pharmacist-led strategies such as renal dose adjustment, individualized risk assessment, and coordinated multidisciplinary care have been shown to enhance anticoagulation outcomes.

Nevertheless, evidence has revealed that pharmacists often report inadequate knowledge of OACs, which may adversely affects their confidence in counselling patients (25, 26). For instance, a study from Middle East found that while pharmacists demonstrated acceptable knowledge levels, many were still less confident in managing OACs therapy effectively (27). Such knowledge gaps may compromise hospital pharmacists’ ability to appropriately counsel patients, as highlighted by a study, in Saudi Arabia, identifying limited training and confidence as key barriers to optimal anticoagulant use (28). Conversely, well-informed pharmacists have the potential to significantly enhance patient safety, improve adherence, and reduce preventable adverse events. Despite this, hospital pharmacists’ involvement in clinical practice across the GCC remains limited. A recent survey involving 64 hospitals in the region reported limited pharmacists involvement in anticoagulant therapy management (29). This underutilisation may contribute to poor medication adherence, increased risk of drug interactions, and preventable harm.

Thus, there remains a significant lack of research focused on the knowledge and practices of hospital pharmacists with regard to OACs in the GCC region. To address this gap, the aim of this scoping review is to explore the knowledge level and practices of hospital pharmacists in the management of oral anticoagulants within hospitals in the GCC countries. Specifically, this review addresses the following research question: What is the knowledge level and practices of hospital pharmacists in OACs dispensing and counselling within the GCC countries?

## 2 Method

This scoping review was conducted according to JBI guidelines and reported in accordance with The Preferred Items for Systematic Reviews and Meta-Analyses extension for Scoping Reviews (PRSIMA-ScR) (30, 31).

### 2.1 Eligibility criteria

The PCC framework (population, concept, and context) was used to determine eligibility criteria. The detailed inclusion and exclusion criteria are presented in Table 1.

**Table 1:** Inclusion and exclusion criteria.

PCC framework	Inclusion	Exclusion
Population (P)	Studies involved licensed hospital pharmacists (any department such as inpatient, outpatient, clinical pharmacy, pharmacy directors etc.,), either alone, or with other healthcare professionals.	Studies did not involve hospital pharmacists or focusing exclusively on community pharmacists, trainees, pharmacy students, or other healthcare professionals.
Concept (c)	Knowledge, awareness, confidence, involvement, and practices regarding the management of OACs including warfarin and DOACs such as apixaban, dabigatran, rivaroxaban, and edoxaban.	Studies that focused solely on injectable anticoagulants. Studies that did not separate pharmacists’ responses from those of other healthcare professionals.
Context (C)	Conducted within healthcare settings in the GCC countries namely Bahrain, Kuwait, Oman, Qatar, Saudi Arabia, and UAE.	Studies conducted outside of the GCC countries.
Type of publication/methodology	Original research articles of any study design, published in any language and any year.	Non-original articles such as review, abstract, case studies and protocols.

### 2.2 Databases

To identify relevant articles, a comprehensive search was conducted across four electronic databases: PubMed, PsycINFO, Scopus, and Web of Science. The search strategy incorporated 3 key themes (1) hospital pharmacists, (2) knowledge and practices, (3) oral anticoagulants. The search strategies for each of the databases are outlined in the Supplementary file.

### 2.3 Search strategy

An initial systematic search was conducted on February 1st 2025, and the search was subsequently re-run on October 28th, 2025. The search strategy was developed in collaboration with an experienced librarian and constructed for each database. Keywords and Medical Subject Headings (MeSH) were employed, along with Boolean operators (OR, AND) and truncations to capture all potentially relevant articles. This search was performed without any restrictions on publication dates to ensure a thorough search of the literature. A weekly literature alert was set up to identify newly published relevant articles, which were screened and included if they met the eligibility criteria. No filters were applied to language, publication date, or study design. The specific keywords used were (*“hospital pharmacist*” OR “clinical pharmacist*”) AND (“*knowledge”* OR “*practice”* OR “*dispensing”* OR “*counselling”* OR “*perception”*) AND (“*warfarin”* OR “*apixaban”* OR “*dabigatran”* OR “*rivaroxaban”* OR “*edoxaban”* OR “oral anticoagulant*”).

All the databases’ results were retrieved and imported into Rayyan and checked for duplicates (32). Rayyan is an online platform designed to facilitate the screening process in a scoping review. After removing all duplicates, titles and abstracts were screened to identify eligible studies, followed by a full-text review to assess eligibility against the predefined inclusion and exclusion criteria. The reference lists of the included studies were also screened to gather additional relevant articles. All authors independently performed the screening, and any disagreements were addressed through discussion until consensus was achieved.

### 2.4 Data extraction

Data from included studies were manually extracted into a Microsoft Word(R) file. The following information was extracted from the included studies: author, publication year, country of origin, study design and instrument, measurement metric, participants, sample size, type of anticoagulants, and findings. Where a study involved several types of pharmacists, only data relevant to hospital pharmacists was extracted.

### 2.5 Synthesis of results

Data from included studies were extracted using charting table and analysed descriptively and presented as narrative synthesis.

### 2.6 Critical appraisal of individual sources of evidence

As this is a scoping review, a formal quality assessment was not conducted.

## 3 Results

**Figure 1:**
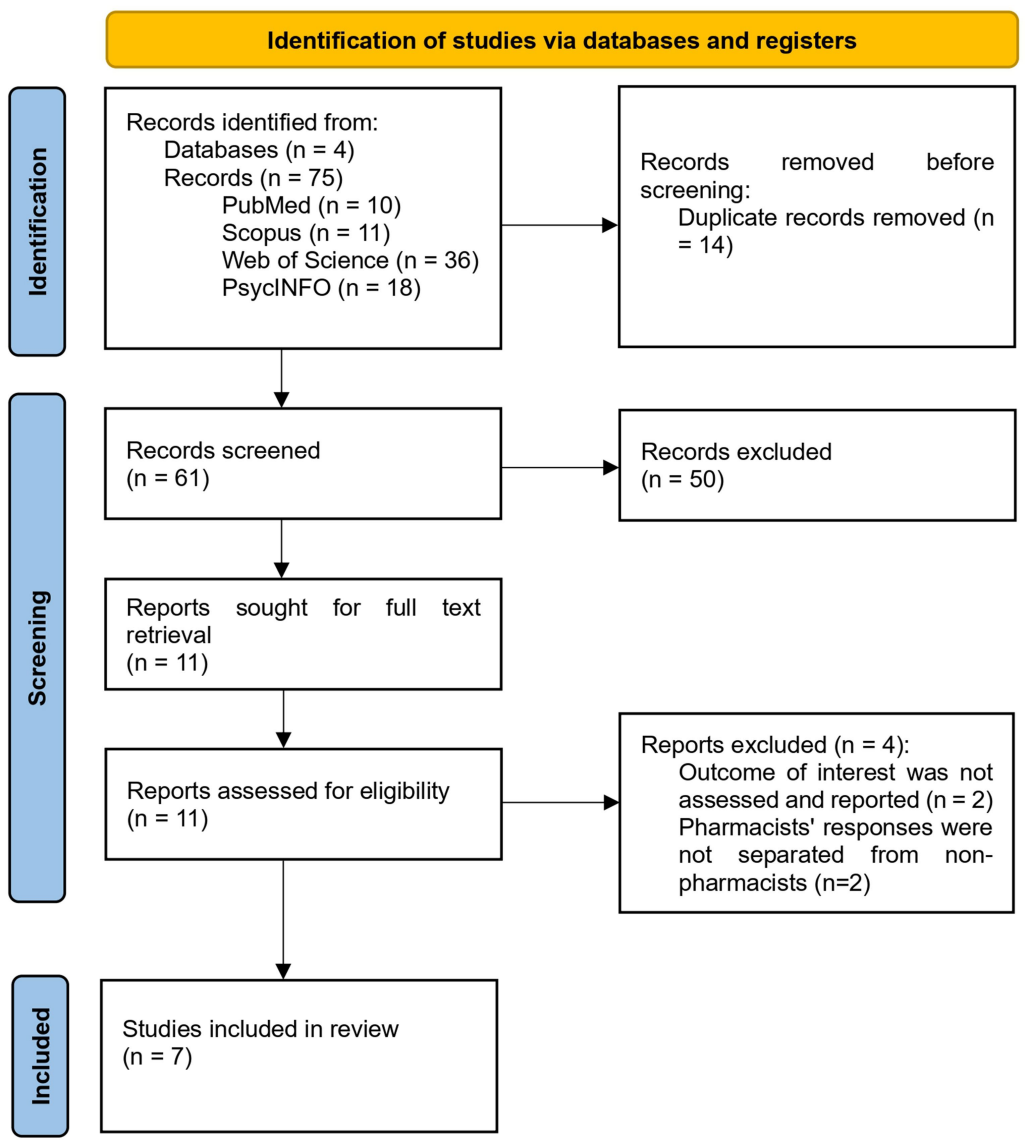
PRISMA flowchart: the study selection.

Figure 1 illustrates the flowchart of the study selection process. The literature search identified 75 potential articles from four databases: PubMed (*n* = 10), Scopus (*n* = 11), Web of Science (*n* = 36), and PsycINFO (*n* = 18). After removing duplicates (*n* = 14), a total of 61 articles were screened for eligibility based on titles and abstracts. Ultimately, 11 articles were retrieved for full-text assessment against the inclusion and exclusion criteria. Four articles were excluded from the review. Two studies did not assess the outcomes of interest (28, 33), and two studies did not report pharmacists’ responses separately from other healthcare professionals (34, 35). As a result, there were 7 articles for final review.

### 3.1 Characteristics of included studies

Seven cross-sectional studies met the inclusion criteria (Table 1). All the studies included hospital pharmacists’ involved in OACs therapy, with sample sizes ranging from 31 to 207 participants. Despite no restriction on publication date, all studies were published between 2016 and 2023. Geographically, the studies were conducted in: Saudi Arabia (*n* = 3), Qatar (*n* = 1), United Arab Emirates (*n* = 1), and across the GCC (*n* = 2). Three studies focused on warfarin, two focused on DOACs, and two studies focused on warfarin and DOACs. All studies used validated questionnaires to obtain their data (Table 2).

**Table 2:** Characteristics of the studies.

Author, year	Country	Study design and Instrument	Measurement metric	Participants	Sample size	Type of anticoagulants	Findings
Al-Arifi et al. (38), 2016	Saudi Arabia	Cross sectional self-administered questionnaire	Level of knowledge	Healthcare providers	31 hospital pharmacists	Warfarin	Inadequate knowledge on warfarin drug/herb interaction. Well-recognized drug interaction with aspirin with warfarin (*n* = 26, 83.9%). Poorly recognized drug interactions: only 4.4% (*n* = 2) identified fluoxetine interaction correctly. Better recognition of herbal interaction: green tea (74% correct answers), and grapefruit (71% correct answers).
Papastergiou et al. (41), 2017	GCC (Kuwait, Qatar, Saudi Arabia, and the UAE)	Cross-sectional international survey	Confidence	Pharmacists	89	Oral anticoagulants	Moderate confidence in counselling patients on OACs therapy. 67% (*n* = 59) of pharmacists reported feeling confident when counselling on VKAs. 49% (*n* = 44) of pharmacists reported feeling confident when counselling on DOACs.
Mansy et al. (39), 2019	Saudi Arabia	Cross sectional self-administered questionnaire	Level of awareness	Hospital pharmacists	116 hospital pharmacists	Rivaroxaban	Clear gaps in knowledge and counselling practices. 50% correctly identified approved uses. 72% felt comfortable counselling. 63% correctly noted adjustments depend on renal function, indication, drug interactions. 75% identified key factors of alternative contraindications (hepatic disease, pregnancy, high bleeding risk, concomitant anticoagulant).
Mohamed et al. (36), 2020	Saudi Arabia	Cross-sectional self-administered questionnaire	Knowledge and counselling practices	Community and hospital pharmacies	97 hospital pharmacists	Warfarin	Moderate knowledge toward warfarin (44.5% overall correct answers). Inappropriate counselling practice (52% average of correct answers). 7.2% relied only on personal knowledge (no clinical resources). Only 11.3% recognized tools (patient booklets, clinical pharmacy clinic follow-ups). 28.6% correctly identified ideal administration time.
El-Bardissy et al. (40), 2020	Qatar	Cross-sectional online questionnaire	Knowledge	Community and hospital pharmacies	161 hospital pharmacists	Direct Oral Anticoagulants	Moderate awareness regarding DOACs (mean score: 41.6% ± 26%). 73% of participants dissatisfied with their DOAC knowledge. Knowledge gaps in: Safe use in renal/hepatic impairment, pregnancy, mechanical heart valves. Monitoring requirements, approved indications, administration, drug–drug interactions.
Al-jedai et al. (42), 2021	GCC (Kuwait, Qatar, Saudi Arabia, Bahrain, and the UAE)	A modified survey questionnaire	Practice	Pharmacist directors	64	Oral anticoagulants	22% of hospitals routinely involve pharmacists in the management of warfarin and DOACs.
Alkherat, and Alkhalidi (37), 2022	United Arab Emirates	Cross-sectional study, online questionnaire	Knowledge and practices	Community and hospital pharmacies	207 hospital pharmacists	Warfarin	Moderate knowledge regarding warfarin (52% of participants). Inappropriate counselling (62.1% of participants). 32% of participants showed poor knowledge. Most hospital pharmacists rarely asked about concurrent herbal medicine use.

### 3.2 Results of individual sources of evidence

Across the seven included studies, the evidence predominantly addressed hospital pharmacists’ knowledge regarding OACs management. Studies also examined counselling practices, self-reported confidence, and hospital pharmacists’ involvement in OACs management. Most outcomes were measured at the individual pharmacist level, with limited assessment of organisational practices in integration of hospital pharmacists into OACs therapy.

### 3.3 Study outcomes

#### 3.3.1 Hospital pharmacists’ knowledge of OACs

Across all studies, pharmacists’ knowledge of OACs ranged from inadequate to moderate, with recurrent deficits identified across both warfarin and DOAC management.

##### 3.3.1.1 Pharmacists’ knowledge of warfarin

Two studies, conducted in Saudi Arabia and the UAE, used the same validated questionnaire to assess pharmacists’ knowledge toward warfarin (36, 37). The questionnaire addressed key warfarin-related topics such as indications, mechanism of action, onset of action, dose adjustment, International Normalised Ratio (INR) monitoring, use during pregnancy and lactation, contraindications, drug interactions, side effects (36, 37).

Overall, hospital pharmacists demonstrated limited knowledge, with overall 44.5% correct answers in the Saudi study (36). Inadequate knowledge was particularly reported on warfarin use during lactation, rare adverse effects, target INR, and dose adjustment (36). Most hospital pharmacists failed to identify significant drug and herb interactions with warfarin, such as allopurinol, sucralfate, estrogen and progestin derivatives, simvastatin, omeprazole, and amoxicillin/clavulanic acid (36).

However, hospital pharmacists showed stronger knowledge in certain areas, including monitoring (94.8% correct answers), pharmacological class of warfarin (75.3% correct answers), and use during pregnancy (85.6% correct answers) (36).

Similarly, findings from the UAE study demonstrated overall moderate knowledge (52% of participants) regarding warfarin (37). Whereas approximately 32% of pharmacists demonstrated poor knowledge of warfarin (37). Marked deficiencies were noted in knowledge of target INR, onset of action, and identification of drug and food interactions (37).

The third study, conducted in Saudi Arabia, investigated hospital pharmacists’ knowledge of warfarin interactions with both drugs and herbal medicines (38). Most pharmacists (*n* = 26, 83.9%) correctly recognised the increased bleeding risk associated with the concomitant use of warfarin and aspirin (38). In contrast, the least recognised interaction was between warfarin and fluoxetine, with only two pharmacists (4.4%) identified it correctly (38). Other poorly recognised interactions included those with omeprazole (*n* = 17), azithromycin (*n* = 14), phenytoin (*n* = 11), and propranolol (*n* = 15) (38). With regard to warfarin–herbal interactions, the majority of pharmacists were able to identify the risks associated with green tea (74% correct answers), grapefruit (71%), garlic (67.7%), and ginkgo biloba (64.5%) (38).

##### 3.3.1.2 Pharmacists’ knowledge of DOACs

Two studies assessed pharmacists’ knowledge of DOACs. A recent study in Saudi Arabia focused specifically on rivaroxaban, using a 15-question self-administered questionnaire (39). The authors assessed hospital pharmacists’ knowledge of rivaroxaban indications, dosing, dose adjustment, monitoring, contraindications, reversal agents, and patient counselling (39). Overall, the study revealed clear gaps in knowledge related to rivaroxaban (39).

Results highlighted weaknesses in several areas. For example, only half of the respondents (50%, *n* = 58) correctly identified its approved uses which involve atrial fibrillation, venous thromboembolism, pulmonary embolism, and post-surgical prophylaxis (39). By contrast, awareness of contraindications was somewhat better, with 75% (*n* = 87) identifying key factors such as hepatic disease, pregnancy, high bleeding risk, and concomitant anticoagulant (38). Knowledge of alternative DOACs varied: 47% identified apixaban, 32.7% were aware of dabigatran, and just 16.4% recognized edoxaban (39). With respect to dosing, 63% correctly noted that adjustments depend on renal function, drug interactions, and indication, but some incorrectly believed that age or weight were determining factors (39).

Despite these gaps, hospital pharmacists demonstrated reasonable awareness of rivaroxaban’s therapeutic role. A large majority 76% (*n* = 88) recognized that rivaroxaban is as effective as warfarin, and nearly all 94% (*n* = 109) realised its advantages, including fewer drug–food interactions, no need for routine monitoring, and reduced risk of intracranial haemorrhage (39).

The other study, conducted in Qatar, used a 25-question Likert-scale survey to assess hospital pharmacists’ knowledge toward DOACs (40). The survey explored four main domains: safety, efficacy, dispensing, and counselling, as well as participants’ satisfaction with their knowledge (40). Overall awareness was moderate, with a mean score of 41.6% ± 26%. Among the domains, awareness of efficacy was the lowest (39.6% ± 29%) (40). These findings aligned with self-satisfaction, as 73% (*n* = 153) of participants reported being dissatisfied with their knowledge of DOACs (40). Specific gaps were noted in pharmacists’ understanding of safe use in patients with renal or hepatic impairment, pregnancy, and mechanical heart valves (40). Further deficits were also identified in key areas such as monitoring requirements, approved indications, administration, and drug–drug interactions (40).

#### 3.3.2 Pharmacists’ practices related to OACs

Counselling practices were assessed in four studies and were generally reported as inadequate or inconsistent. In the Saudi warfarin study, 7.2% of hospital pharmacists relied solely on previous knowledge without using clinical resources when counselling their patients (36). This may explain the inadequate counselling practices observed, where only 28.6% of responses correctly identified the ideal time of warfarin administration and 32.1% provided correct instructions on missed doses (36). Furthermore, adherence support tools such as patient booklets or clinical pharmacy clinic follow-ups were identified by only 11.3% of pharmacists (36).

Similarly, in the UAE study, moderate counselling practice were reported (62.1% of participants) and 16% showed poor performance in counselling practice (37). Moreover, the authors noted that most hospital pharmacists rarely asked patients if they were consuming concurrent herbal medicines (37).

In the rivaroxaban study, Counselling-related knowledge was also inconsistent (39). Almost half of respondents (*n* = 58) answered incorrectly or were uncertain about whether rivaroxaban can be taken with food or if withheld before invasive surgery (39). When assessing patient counselling, almost three-quarters of participants (72%, *n* = 84) reported feeling comfortable in counselling patients regarding rivaroxaban (39). However, those who felt uncomfortable attributed it to lack of knowledge in key areas such as indications, dosing, drug interactions, dose adjustments, and the availability of an antidote (39). Similarly, the study conducted in Qatar reported moderate levels of counselling practice related to DOACs therapy, with a mean score of 44.4% ± 36% (40).

Across studies, limited use of structured counselling tools, such as patient booklets or anticoagulation clinic follow-up systems, was evident, indicating gaps in patient education support process.

#### 3.3.3 Pharmacists’ confidence in OACs-related care

Within this context, an international cross-sectional survey explored pharmacists’ confidence in counselling patients on OACs therapies, specifically comparing VKAs with the newer DOACs (41). The research included 89 pharmacists from the GCC countries namely Kuwait, Qatar, Saudi Arabia, and the UAE representing 2.1% of the total study sample (41). Overall, the GCC pharmacists demonstrated a moderate level of confidence in providing OACs-related pharmaceutical care (41). Approximately 67% (*n* = 59) of respondents reported feeling very confident or confident when counselling on VKAs, reflecting their familiarity with these long-established therapies (41). In contrast, only about 49% (*n* = 44) expressed similar confidence when advising on DOACs, highlighting a gap in experience with these newer medicines (41).

#### 3.3.4 Pharmacists’ involvement in OACs management

A multinational study conducted across the GCC countries explored hospital pharmacists’ involvement in OACs management (42). The findings revealed a variability in practice across hospitals in the GCC (42). Approximately 22% (*n* = 14) of hospitals reported that pharmacists routinely managed the dosing and monitoring of OACs therapy (42). In contrast, 48% (*n* = 31) indicated that pharmacists were not involved in warfarin management, while 29% (*n* = 19) reported that pharmacists managed warfarin therapy upon request (42).

On the other hand, Al Jedai et al. reported similar findings regarding pharmacists’ involvement in the management of DOACs (42). The study revealed that only 22% (*n* = 14) of hospitals across the GCC had pharmacists routinely engaged in DOAC dosing and monitoring (42). In contrast, more than half of the hospitals (51%, *n* = 33) reported no pharmacist involvement in these activities, while 26% (*n* = 17) indicated that pharmacists participated in DOAC management only upon request (42).

## 4 Discussion

To our knowledge, this is the first scoping review to assess the existing evidence on hospital pharmacists’ knowledge and practices related to OACs management in the GCC countries. A total of seven cross-sectional studies were included. These studies focused mainly on warfarin, with limited coverage of DOACs. Hospital pharmacists demonstrated moderate understanding of warfarin’s administration and pharmacological class. However, marked knowledge deficits were observed in critical areas such as warfarin use during pregnancy, drug-food and herb interactions, and INR monitoring. Similarly, their understanding of DOACs was limited, particularly concerning bleeding risks and dose adjustments. Collectively, these findings highlight interconnected themes of pharmacists’ knowledge, practice, and differential familiarity with warfarin versus DOACs, which together shape pharmacists’ involvement in OACs management in the GCC.

Most studies included in this review focused on warfarin, reflecting its long history of clinical use. However, the focus on DOACs was very limited, even though these agents have increasingly replaced warfarin worldwide, including in the GCC countries. This may reflect various factors, such as earlier familiarity with warfarin or slower integration of DOACs into clinical practice. Although DOACs were firstly approved in the European Union in 2008 (43), their inclusion in hospital formularies and widespread use as alternatives to warfarin will require additional time. Such delays are further compounded at international level, including within the GCC countries where variations in regulatory pathways, healthcare infrastructure, and local clinical guidelines can slow adoption into routine practice. Nevertheless, this explanation seems less convincing, as recent evidence indicates a growing trend in DOAC use across the GCC. A more likely explanation is that research efforts have not yet aligned with evolving clinical practice, leaving gaps in addressing hospital pharmacists’ roles in managing these newer oral anticoagulants.

Taken together, the findings of this review indicate that hospital pharmacists in the GCC countries possess inadequate knowledge and practices of OACs therapy. They exhibit persistent deficits in clinically complex areas, particularly drug interactions, dose adjustments, and special populations. Counselling practices are frequently suboptimal, despite moderate self-reported confidence. Moreover, hospital pharmacists’ involvement in OACs management remains limited and inconsistent across the GCC.

### 4.1 Pharmacists’ knowledge of oral anticoagulants

Hospital pharmacists play a pivotal role in the safe and effective use of OACs, and their performance is directly influenced by their level of knowledge. Previous research has shown that pharmacists identified inadequate knowledge as a main obstacle for effective OACs management (44). Consistent with this evidence, this review found that hospital pharmacists in the GCC exhibit gaps in knowledge that may hinder optimal oral anticoagulant management. Many hospital pharmacists lacked understanding of how to adjust OACs doses appropriately according to INR values (warfarin), renal function, or clinical indication. Some even incorrectly believed that factors such as age or weight were required for rivaroxaban dosing. This lack of understanding is critical, as inappropriate dose adjustments can lead either to subtherapeutic anticoagulation and thromboembolic events, or to overdosing with increased bleeding risk.

In addition, knowledge gaps extended to DOACs safe use in pregnancy, lactation, renal and hepatic impairment, and in patients with mechanical heart valves. These populations represent high-risk groups in which inappropriate anticoagulant selection or dosing may cause serious maternal, fetal, or patient harm. In contrast, pharmacists demonstrated stronger knowledge of warfarin regarding monitoring, pharmacological classification, and use during pregnancy. This may be attributed to the long use of warfarin in clinical practice and the emphasis placed on its management in undergraduate study and continuing education across regions. Pharmacists may also more familiar with warfarin’s safety profile and often pay a close attention to its risks in vulnerable populations, such as pregnant women. Conversely, their limited awareness of similar safety issues related to DOACs may reflect the relative novelty of these medications and variability in clinical experiences. Nevertheless, direct comparisons between studies should be interpreted cautiously due to differences in hospital pharmacists’ experience, healthcare settings, local guidelines, and medications availability.

Drug and herbal interaction knowledge with OACs were particularly concerning. Hospital pharmacists frequently failed to recognize interactions of warfarin with commonly prescribed medications (e.g., fluoxetine, omeprazole, azithromycin, simvastatin, amoxicillin/clavulanic acid, and phenytoin), though bleeding risk with aspirin was well recognized. Given that these medicines may commonly prescribed to patients who use warfarin, it would have been reasonable to expect hospital pharmacists to be aware of these specific interactions. For instance, evidence indicates a high prevalence of dyslipidaemia in Saudi Arabia, which often requires patients to be prescribed statins such as simvastatin (45, 46). Therefore, the inability to identify interactions between warfarin and commonly used drugs like simvastatin raises concerns regarding hospital pharmacists’ applied pharmacotherapy knowledge. Unrecognized serious interactions can directly lead to therapeutic failure or life-threatening bleeding and so the reasons for not recognizing these interactions and other knowledge gaps needs exploring.

The differences in hospital pharmacists’ knowledge of warfarin drug and food interactions observed across studies may reflect variations in institutional contexts, professional training, and patient populations. Pharmacists working in tertiary care hospitals, for example, often receive more extensive clinical exposure and continuing education on oral anticoagulant management than those in smaller hospitals. Furthermore, regional differences in the prevalence of certain diseases may expose pharmacists to distinct medication profiles, thereby influencing their experiential familiarity with relevant drug interactions.

These findings align with studies outside the GCC. Research in the United States and Australia highlighted similar gaps, particularly in warfarin’s interactions with food, nutrients, and complementary medicines (47, 48). Another study in Iran revealed low to moderate knowledge among pharmacists toward DOACs (27). Conversely, other evidence shows more encouraging trends. In contrast, a survey in Croatia reported strong pharmacists’ knowledge on warfarin interactions, with almost 94% of pharmacists recognizing the interaction between warfarin and green leafy vegetables (49). Differences across countries may be attributable to variations in educational preparation, clinical practice patterns, and the relative prevalence of warfarin compared with DOACs.

### 4.2 Pharmacists’ practices of oral anticoagulants

Knowledge deficits can influence practice. This was clearly reflected in the included studies, where hospital pharmacists’ counselling practices were consistently inadequate. Hospital pharmacists frequently failed to provide accurate advice on missed doses, administration timing, or perioperative precautions, and they rarely inquired about patients’ use of herbal medicines. Limited awareness of adherence tools such as patient booklets or follow-up clinics may undermines continuity of care as these tools are designed to support ongoing monitoring, reinforce patients’ education, and consistent follow up. Given that OACs are high-risk medicines requiring strict adherence, insufficient counselling increases the likelihood of medication errors, poor adherence, and adverse outcomes.

These findings are not unique to the GCC. A global survey indicated that many pharmacists lack sufficient knowledge to confidently provide counselling on OACs (50). Moreover, another international survey covering 22 countries, including the GCC countries, revealed that renal dose adjustments and patient counselling on OACs were performed in only 75% of the surveyed settings (51). Similarly, a large U.S. based study involving 1,350 healthcare facilities found limited pharmacist involvement in patient education for OACs (52). Collectively, these findings highlight that the practice gaps observed in the GCC reflect systemic challenges rather than regional or institutional malpractice.

Several studies have found that pharmacists report higher confidence when counselling on warfarin compared to DOACs (53, 54), with DOAC counselling often identified as the area of lowest self-reported confidence (54). A possible explanation is that DOACs were only introduced to the market in 2010 and are therefore relatively new compared to warfarin, which has been in use since the 1950s. Consequently, the higher confidence observed in warfarin counselling may be attributed to the greater clinical experience and longer familiarity with this medication compared to the more recently introduced DOACs. In contrast, a study from Czech Republic (Czechia) reported that majority of pharmacists felt confident toward DOACs which is unsurprising, since almost 86% of participants (*n* = 139) were regularly involved in DOAC dispensing in their daily practice (55). These differences might also be explained by the variations of the training and roles of pharmacists in this area. For instance, in some countries hospital pharmacists may receive structured training programs, continuing education opportunities, or have an expanded clinical role in OACs management, which can improve their confidence in counselling patients on DOACs. In other contexts, where training is limited or hospital pharmacists’ responsibilities are primarily focused on dispensing rather than direct patient counselling, confidence levels may remain lower. However, such deficiencies in counselling practice could compromise patient safety and therapeutic outcomes, particularly in populations at high risk for adverse events from oral anticoagulants.

Despite their vital role, hospital pharmacists involvement in the management of OACs varies significantly across different countries and healthcare settings worldwide, influencing their direct impact on OACs management. In developed healthcare systems such as the UK, hospital pharmacists are often integrated into anticoagulation services, where they are responsible not only for dispensing but also for dose adjustment, switching between DOACs, INR monitoring, renal dosing, bleeding risk assessment, and patient education (56). Pharmacist-led anticoagulation clinics are a key feature of care pathways in National Health Service (NHS) settings (56). Evidence from multinational surveys indicated that UK pharmacists showed higher confidence in managing OACs compared to pharmacists from other countries (41). This finding is supported by a recent study indicating that UK pharmacists reported adequate knowledge in OACs counselling, with most relying on sources such as National Institute for Health and Care Excellence (NICE) and the British National Formulary (BNF) to support patients’ consultation (5). This highlights the potential benefits of integrating pharmacists more fully into anticoagulation management internationally, supported by appropriate education and practice. In contrast, when compared with practices in GCC countries, hospital pharmacists’ clinical role remains largely focused on dispensing without formal authority to initiate or modify therapy. This restricted scope of practice may partly explain the differences observed in pharmacists’ knowledge and confidence compared with findings from other countries.

Nevertheless, the scope of pharmacy practice is currently undergoing a significant transformation worldwide, including in Saudi Arabia. The emerging role of clinical pharmacists has demonstrated effectiveness across a wide range of interventions in diabetes, dyslipidaemia, hypertension, antimicrobial stewardship, medication adherence, and medication reconciliation (57, 58).

With respect to OACs, evidence from the literature showed that clinical pharmacists contributed significantly to the appropriate selection, dosing, review, and patient counselling related to OACs therapy (59, 60). Clinical pharmacist involvement has been associated with improved therapeutic outcomes, including better anticoagulation control and fewer discharge delays (61). In Saudi Arabia, evidence indicates that clinical pharmacist involvement increases the appropriate use of apixaban after discharge without increasing the risk of bleeding or other adverse events (62). Furthermore, another study demonstrated that clinical pharmacist interventions can improve the health-related quality of life of patients receiving warfarin therapy (63).

In response to this growing evidence, the Saudi Commission for Health Specialties has developed programmes and clinical pathways to support the role of clinical pharmacists in OAC management and to promote greater integration of clinical pharmacists within multidisciplinary healthcare teams (64, 65). These findings support integrating clinical pharmacists into anticoagulation management services to improve patient safety and therapeutic outcomes. Nevertheless, the roles and responsibilities of clinical pharmacists remain inconsistent across healthcare settings in Saudi Arabia (66), highlighting the need for targeted initiatives and policy reforms to expand and standardise clinical pharmacists’ involvement in OACs management.

Overall, this scoping review reveals several consistent patterns across the included studies. Knowledge deficits were evident across OACs classes, although they were more pronounced for DOACs than for warfarin, particularly in areas related to dosing adjustments, safety in special populations, and bleeding risk management. Moreover, the findings suggest that hospital pharmacists’ knowledge, clinical practices, and familiarity with specific OACs are closely interrelated. Greater familiarity with warfarin which driven by its long clinical use, educational emphasis, and structured monitoring requirements, appears to contribute to relatively stronger knowledge and counselling confidence compared with DOACs. In contrast, limited exposure to DOACs, coupled with variability in training and clinical involvement, may contribute to deficits in knowledge, inadequate counselling confidence, and suboptimal practice.

### 4.3 Implications for practice

The collected findings of this review highlight the need to improve hospital pharmacists’ knowledge and practices related to OACs management. This apparent lack of knowledge may be due to inadequate or insufficient training. Therefore, it is important to first examine the type of training pharmacists have received in this area. Based on these insights, targeted training programmes might be implemented to address the identified knowledge gaps. These programmes could strengthen hospital pharmacists’ ability to confidently perform clinical tasks and support the safe and effective use of OACs. Enhancing hospital pharmacists’ knowledge in this area can also improve patient understanding and satisfaction when receiving counselling from well-trained hospital pharmacists. Creating consistent framework such as standard operating procedures for patient counselling may standardise and minimise variability in counselling practices. In turn, this can contribute to better therapeutic outcomes and adherence. Policymakers should recognise the value of investing in structured training initiatives and integrating them into hospital-based continuing education systems.

### 4.4 Limitations

However, several limitations must be acknowledged. First, the geographic scope is somewhat restricted, and the majority of the studies identified originated from Saudi Arabia, Qatar, and UAE. There were no studies from Oman, Kuwait, or Bahrain, which limits the generalizability of the findings across the entire GCC, therefore further work is needed to explore OACs dispensing and counselling practices in these countries. Second, all included studies used cross-sectional designs and self-reported data, which may introduce bias. In many cases, participants represented a mix of healthcare professionals, with no studies exclusively focusing on hospital pharmacists, despite their frontline role in OACs management in the GCC region. Additionally, several studies had small sample sizes and were conducted in urban settings, with no representation from rural healthcare facilities. This is very important, as rural areas in the GCC host a substantial number of healthcare institutions that constitute an essential component of the region’s healthcare system. This urban-centric approach further limits the applicability of findings to diverse practice environments. Ultimately, only a limited number of studies were identified, highlighting the overall paucity of research in this field.

## 5 Conclusion

This review demonstrates that hospital pharmacists in the GCC are inconsistently involved in OACs management, and their knowledge and practices are inadequate. The studies included in this review reveal gaps in hospital pharmacists’ knowledge and counselling practices related to both warfarin and direct oral anticoagulants, with clear implications for patient safety and therapeutic effectiveness. To address these gaps, the scope of pharmacy practice should be clarified, and efforts may focus on improving hospital pharmacists’ skills through clinical training, continuing education, and greater integration within multidisciplinary anticoagulation teams. Ultimately, empowering hospital pharmacists in the GCC to take a more active role in OACs management has the potential to improve patient safety and outcomes significantly.

Further research exploring hospital pharmacists’ experiences, practice challenges, and training needs is essential. Insights from such studies can guide the development of targeted educational programs and support policies that expand hospital pharmacists’ role, ultimately strengthening OACs management and patient care within the GCC and beyond.
