# Supplementary material for: Exploring the knowledge level and practices of hospital pharmacists in management of oral anticoagulants in Gulf Cooperation Council countries: a scoping review of literature
Source: Front Public Health. 2026 Jan 22;14:1743611. doi: 10.3389/fpubh.2026.1743611 (PMC12872889; doi:10.3389/fpubh.2026.1743611)
Supplement: Supplementary file 1 [file Table_1.DOCX]

Supplementary Material S1

Database: PubMed

Date of search: February 1, 2025

(((pharmacists[mesh] OR Pharmacists[tiab] OR “Medication specialist*”[tiab] OR "Pharmacy Technicians"[Mesh] OR "pharmacy technicians"[tiab]) AND ("Role"[Mesh] OR Knowledge OR attitude OR Educat* OR awareness OR understanding OR counselling OR "counselling practices" OR beliefs OR viewpoints OR "Practice pattern" OR "Counseling"[Mesh] OR "clinical practice" OR responsibility OR Confide* OR Perception OR concept OR "self-esteem" OR "Self-Concept"[Mesh] OR experience*[tiab] OR "Awareness"[Mesh] OR "Attitude"[Mesh])) AND (Warfarin OR Coumadin OR "Direct oral anticoagulants" OR "DOAC" OR rivaroxaban OR edoxaban OR apixaban OR dabigatran OR "Vitamin K antagonists" OR "oral direct thrombin inhibitor" OR anticoagula* OR "direct factor Xa inhibitor" OR "Factor Xa Inhibitors"[Mesh] OR "novel oral anticoagulants" OR "NOAC" OR "Antithrombins"[Mesh])) AND (GCC OR "gulf countries" OR "gulf cooperation council" OR "GCC countries" OR Saudi OR "Saudi Arabia"[MeSH] OR Qatar OR Kuwait OR "United Arab Emirates" OR "UAE" OR Oman OR Bahrain OR Riyadh OR Arar OR Khobar OR Dammam OR Alhassa OR "eastern region" OR Jeddah OR Taif OR "abu dhabi" OR Dubai OR Doha)

Database: Web of science

Date of search: March 23, 2025

((Pharmacists OR “Medication specialist*” OR "Pharmacy Technicians") AND (Role OR Knowledge OR attitude OR Educat* OR awareness OR understanding OR beliefs OR viewpoints OR "Practice pattern" OR "clinical practice" OR responsibility OR Confide* OR Perception OR concept OR "self-esteem" OR "Self-Concept") AND (Warfarin OR Coumadin OR "Direct oral anticoagulants" OR "DOAC" OR rivaroxaban OR apixaban OR dabigatran OR edoxaban OR anticoagula* OR "novel oral anticoagulants" OR "Vitamin K antagonists" OR "oral direct thrombin inhibitor" OR "direct factor Xa inhibitor" OR "Factor Xa Inhibitors" OR "Antithrombins") AND (GCC OR "gulf countries" OR "gulf cooperation council" OR "GCC countries" OR Saudi OR "Saudi Arabia" OR Qatar OR Kuwait OR "United Arab Emirates" OR "UAE" OR Oman OR Bahrain OR Riyadh OR Khobar OR Jeddah OR Taif OR Dammam OR Dubai OR Alhassa OR "eastern region" OR Doha OR "abu dhabi" OR Arar))

Database: Scopus

Date of search: July 26, 2025

(( pharmacists OR pharmacis* OR {Pharmacy Technicians} OR {medication specialist} OR {medication specialists} ) AND ( role OR knowledge OR attitude OR {Knowledge and attitude} OR educat* OR {self-esteem} OR {Self-Concept} OR understanding OR practice OR {clinical practice} OR responsibility OR confide* OR awareness OR counselling ) AND ( warfarin OR coumadin OR anticoagula* OR edoxaban OR {NOAC} OR {novel oral anticoagulants} {Direct Oral Anticoagulants} OR {DOAC} OR rivaroxaban OR apixaban OR dabigatran OR {Vitamin K antagonists} OR {oral direct thrombin inhibitor} OR {direct factor xa inhibitor} OR {factor xa inhibitors} OR antithrombins ) AND ( gcc OR {gulf countries} OR {gulf cooperation council} OR {GCC countries} OR saudi OR {Saudi Arabia} OR qatar OR kuwait OR {United Arab Emirates} OR {UAE} OR oman OR bahrain OR riyadh OR khobar OR jeddah OR taif OR dammam OR dubai OR alhassa OR {eastern region} OR doha OR {Abu Dhabi} OR arar ) )

Database: PsycInfo

Date of search: October 12, 2025

((Pharmacists or Medication specialist or Pharmacy Technicians) and (Role or Knowledge or attitude or Education or awareness or understanding or beliefs or viewpoints or Practice pattern or clinical practice or responsibility or Confident or Perception or concept or self esteem or Self Concept) and (Warfarin or Coumadin or Direct oral anticoagulants or rivaroxaban or apixaban or dabigatran or Anticoagulants or novel oral anticoagulants or Vitamin K antagonists or oral direct thrombin inhibitor or direct factor Xa inhibitor or Factor Xa Inhibitors or Antithrombins) and (GCC or Gulf countries or Gulf Cooperation Council or GCC countries or Saudi or Saudi Arabia or Qatar or Kuwait or United Arab Emirates or UAE or Oman or Bahrain or Riyadh or Khobar or Jeddah or Taif or Dammam or Dubai or Eastern Region or Doha or Abu Dhabi or Alhassa or Arar))

Additional step: Manual screening of reference lists of all included studies and relevant reviews.
